# A boundary delimitation algorithm to approximate cell soma volumes of bipolar cells from topographical data obtained by scanning probe microscopy

**DOI:** 10.1186/1471-2105-11-323

**Published:** 2010-06-15

**Authors:** Patrick Happel, Kerstin Möller, Ralf Kunz, Irmgard D Dietzel

**Affiliations:** 1 Central Unit for Ionbeams and Radionuclides (RUBION), Ruhr University of Bochum, D-44780 Bochum, Germany; 2 Department of Molecular Neurobiochemistry, Ruhr University of Bochum, D-44780 Bochum, Germany; 3 Department of Cardiovascular Physiology, University of Heidelberg, Heidelberg, Germany

## Abstract

**Background:**

Cell volume determination plays a pivotal role in the investigation of the biophysical mechanisms underlying various cellular processes. Whereas light microscopy in principle enables one to obtain three dimensional data, the reconstruction of cell volume from *z*-stacks is a time consuming procedure. Thus, three dimensional topographic representations of cells are easier to obtain by scanning probe microscopical measurements.

**Results:**

We present a method of separating the cell soma volume of bipolar cells in adherent cell cultures from the contributions of the cell processes from data obtained by scanning ion conductance microscopy. Soma volume changes between successive scans obtained from the same cell can then be computed even if the cell is changing its position within the observed area. We demonstrate that the estimation of the cell volume on the basis of the width and the length of a cell may lead to erroneous determination of cell volume changes.

**Conclusions:**

We provide a new algorithm to repeatedly determine single cell soma volume and thus to quantify cell volume changes during cell movements occuring over a time range of hours.

## Background

Cell volume regulation occurs in a wide variety of tissues from kidney to brain [1-4]. Although much is known about ion and water fluxes involved in many regulatory processes, no method has so far been designed to investigate potential volume changes in moving cells. Light microscopy enables one to estimate the cellular volume via different techniques, ranging from extrapolation on the basis of the width and length of the cell [5], changes in light intensity and light scattering [6,7], various staining techniques [8,9] to quantitative phase microscopy [10]. All these techniques fail when it is required to investigate the volume of a cell undergoing notable changes in shape such as occur during cell migration [11,12] since they require constant parameters such as height or refractive index and some have additional disadvantages such as bleaching of the dye [13].

A promising approach to circumvent these problems is to measure volume directly with a scanning probe microscope. Direct measurements of cellular volume have been performed by scanning ion conductance microscopy (SICM) on cellular layers [14] and single cells [15,16] and by atomic force microscopy (AFM) on living and fixed cells [17,18]. The volume determined by SICM of cells forming a confluent layer has been validated by scanning confocal laser microscopy [14].

**Figure 1:**
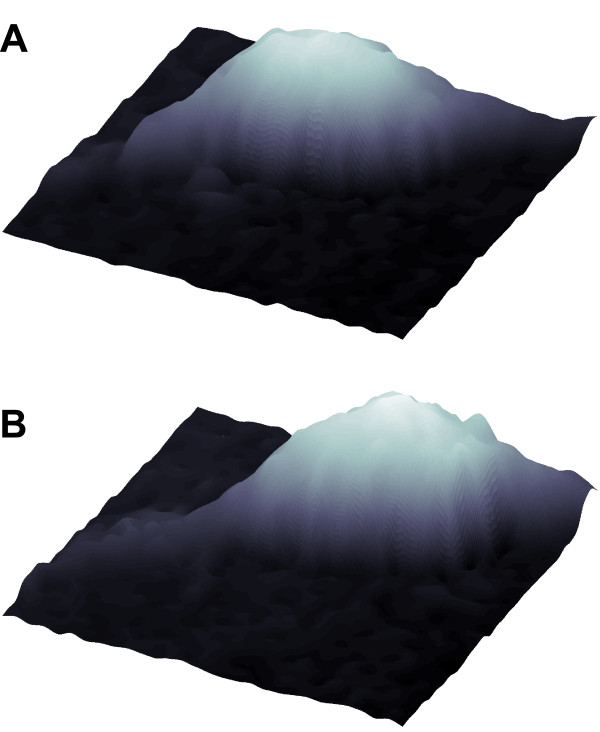
**An oligodendrocyte precursor cell undergoing temporal changes in position within the observed scanning frame**. A and B: Scanning ion conductance microscopic images of the same cell obtained at *t *= 0 minutes and *t *= 10 minutes. Note the change in position of the cell body. Due to the dislocation of the cell soma the two scans include variable amounts of the processes extending from the soma. To obtain a quantification of the cell soma volume, an algorithm was developed to separate the soma from the processes.

Volume determination by scanning probe microscopy assumes that cells are closely attached to the substrate and is mostly based on the height of every observed pixel [14,15,17,18]. When trying to investigate the volume dynamics of the somata of bipolar cells such as oligodendrocyte precursor cells (OPCs), the dimensions as well as the lateral resolution of the scan have to be restricted in order to obtain an acceptable temporal resolution. For the investigation of neural cells exhibiting long extensions most scanning frames inevitably crop the extended cellular ramifications. This leads to errors in the volume determination of migrating cells since the fraction of the processes located within the scanning frame may vary in successively obtained recordings, as for the process located on the right side in Figure 1.

We have previously proposed a method for distinguishing between cellular somata and processes during investigations of the surface of oligodendrocyte cell bodies at different developmental stages [19]. Here, every pixel exceeding a certain height was assigned to the cell soma. In cells undergoing marked changes in shape this method fails since it would result in different soma volumes if a cell flattens but performs a compensatory widening thus maintaining its volume. Hence, to estimate volume changes of single bipolar cell somata changing their shape and position we have now developed a novel procedure that allows us to separate the cell soma volume from the extended peripheral membrane processes of bipolar cells.

## Results and Discussion

**Figure 2:**
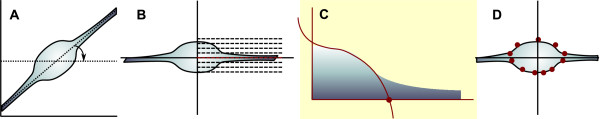
**Principles of the approximation procedure to determine the basal soma area**. A: The heading direction of the bipolar cell within the observed area is estimated, indicated by the arc. B: The cell is rotated around its highest part (represented by *C*_90 _as defined in the text, see also Figure 3) by its heading direction to position the cell parallel to the abscissa. The cell is divided into its frontal and its rear part at the level of *C*_90_. Each part of the cell is investigated linewise as indicated by the dashed lines in B. C: Side view (emphasized by the yellow box) of a single line. The contour of the cell at a single line is approximated by fitting a polynomial to the cell. The root of the polynomial (red dot in C) yields the boundary of the cell soma for the particular line. D depicts the result of the approximation procedure: The roots (red dots) obtained from fitting every single line of the frontal and rear part of the cell approximate the boundary of the cell soma.

The boundary delimitation algorithm (BDA) for approximating the basal area of the cell soma of bipolar cells was divided into four steps as depicted in Figure 2. OPCs in culture generally have two long processes at opposite poles of an ellipsoidal soma, and move in the direction of one of them. Whereas a single image does not allow the identification of the direction of movement it still allows the determination of the direction of the processes. We call this the "heading direction" of the cell. As the first step of the BDA the heading direction of the bipolar cell, indicated by the angle drawn in Figure 2A, was estimated and subsequently the cell rotated in order to position the heading direction of the cell parallel to the abscissa (Figure 2B). Second, the cell was divided into its front and rear parts at the level of the nucleus. Third, starting at the nucleus, the contour of the soma was approximated by linewise (as indicated by the dashed lines in Figure 2B) fitting of polynomials to the data for the frontal and the rear parts of the cell separately. The root of the fit for every single line, indicated by the red dot in Figure 2C, was used to delimit the cell soma from the cell processes (Figure 2D). A compressed archive of the Matlab functions used to perform the BDA as detailed in the following is available as Additional File 1.

### Approximation of the position of the nucleus

Atomic force microscopy measurements on hippocampal neurons revealed that the higher parts of the cell body form a harder structure and correspond most likely to the nucleus [20]. In order to determine a single point that represents the location of the nucleus the following procedure was employed: We stained the nucleus using Hoechst 33342 dye and recorded an epifluorescence as well as a phase contrast image.

Subsequently an SICM scan was performed and the relative position of the SICM scan was determined within the micrograph [19]. We then investigated the distance of the centroid of different horizontal sections through the SICM scans to the centroid of the Hoechst-stained nucleus. The horizontal sections consisted of the areas that were covered by pixels *P*_*i *_= (*x*_*i*_, *y*_*i*_, *z*_*i*_) (with *i *denoting the number of the pixel) exceeding a certain height *T z*_max_, where *T *denotes a predefined threshold and *z*_max _denotes the maximum cell height. To calculate the position of the centroid *C*_*T *_we reduced the *z*-coordinates of *P*_*i *_to boolean values *z*_*T,i *_= [*z*_*i *_>*T z*_max_]. The square brackets indicate a Heaviside-like function that yields 1 if the enclosed condition is true and 0 otherwise [21,22]. Furthermore, we assumed constant step sizes between the pixels and thus calculated the *x*-coordinate of *C*_*T*_, , as:(1)

 was calculated in the same manner.

We next investigated the distance between *C*_*T *_and the centroid of the Hoechst 33342 staining of the nucleus (see Methods section) for various thresholds *T*.

**Figure 3:**
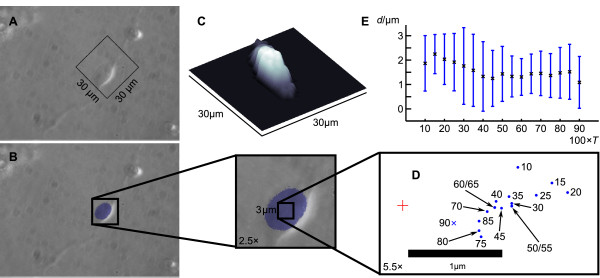
**Representation of the location of the nucleus by *C*_90_**. A and B show light microscopic images from an oligodendrocyte precursor cell whose nucleus was stained using Hoechst 33342 (B) and that was scanned by backstep SICM (C). The position of the scan is depicted in A and a three dimensional representation of the data obtained by SICM is shown in C. The positions of *C*_*T *_for varying *T *(between 10% and 90% of the maximal *z*-value) calculated from the SICM data with respect to the position of the centroid of the stained area (obtained from fluorescence microscopy as shown in B, marked by the red cross-hair) are drawn rotated and magnified in D (blue dots and blue cross, labels indicate *T *in percent). E shows the average distances between *C*_*T *_and the centroid of the staining of the nucleus obtained from 3 different determinations, error bars indicate ± SD.

Figure 3A-C show the phase contrast, epifluorescence and SICM image of an OPC. The position of the SICM scan within the light microscopic image is depicted as the black square in Figure 3A. The positions of *C*_*T *_for *T *= 0.1, 0.15,..., 0.9 and the centroid of the nucleus obtained from the epifluorescence staining (*C*_fluo_) are depicted in Figure 3D. *C*_90 _(note that we use *T *in percent when indexing or labeling, thus *C*_*T *= 0.9 _≡ *C*_90_) exhibited the minimal distance to *C*_fluo_. Figure 3E shows the average distances between *C*_*T *_and *C*_fluo _obtained from three different recordings. This confirms that *C*_90 _is located closest to *C*_fluo_. Note that representations determined by using a larger threshold such as *C*_95 _often base on disjunct areas and were not investigated in detail. Thus we used *C*_90 _to approximate the position of the nucleus in the following.

### Estimation of the heading direction of the cell

**Figure 4:**
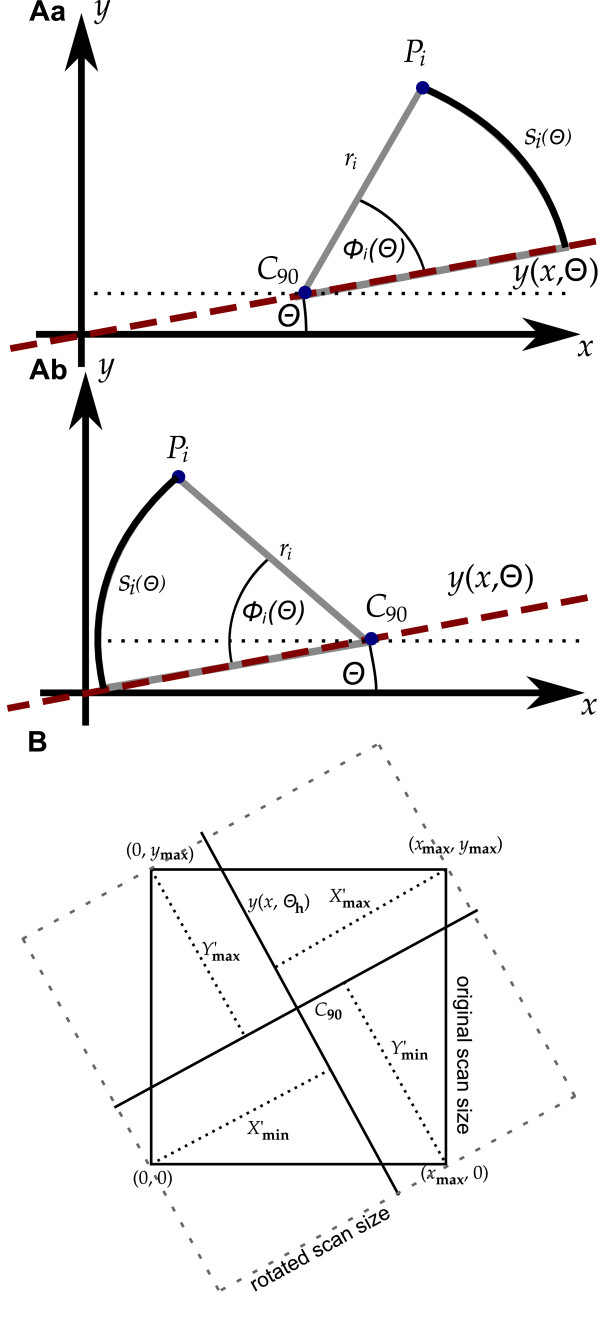
**Overview of the various lengths, angles and points**. A: The angle Θ defines the direction of a straight line *y *(*x*, Θ) through *C*_90_. The angle *ϕ*_*i *_(Θ) originates at *C*_90 _and is defined as the smallest angle between the line *r*_*i *_from *C*_90 _to the pixel *P*_*i *_and *y *(*x*, Θ). Aa and Ab illustrate the relation for *P*_*i *_located at opposite sides of *C*_90_. *s*_*i*_(*ϕ*) is defined as the arc of the circle with radius *r*_*i *_from *y *(*x*; Θ) to *P*_*i *_. B: The dimensions of the translated and rotated scan data based on the distances (dotted lines) of the vertices of the original scan to the straight lines through *C*_90 _in and perpendicular to the heading direction of the cell (straight lines). Note the increase in basal area caused by the rotation (see also Figure 11 and Figure 12).

OPCs display a bipolar phenotype terminating in two cell processes that are most commonly originating from the opposite ends of the cell soma. This enables one to approximate the heading direction *θ*_h _of an OPC by rotating a straight line(2)

through *C*_90 _as the approximation of a straight line through the nucleus. In order to determine the heading direction of the cell we considered the arcs from each pixel representing the cell to *y*(*x*, *θ*) Let *ϕ*_*i*_(*θ*) denote the smallest angle between *P*_*i *_and *y*(*x*, *θ*) and *r*_*i *_denote the distance from *C*_90 _to *P*_*i*_. Then the length *s*_*i*_(*θ*) of the corresponding arc is calculated as *s*_*i*_(*θ*) = *ϕ*_*i*_(*θ*)*r*_*i*_. Figure 4A illustrates the relations between the introduced angles, lines and points for two different pixels *P*_*i *_located at opposite sides of *C*_90_. We now defined the angle *θ*_h_, that minimized the sum of *s*_*i *_(*θ*) and thus matched the condition(3)

as the heading direction of the cell. Here we assumed that pixels that exhibited a height of ≤1 μm represented the cell culture dish rather than the cell. Equation (3) was solved numerically by testing all angles 0 ≤ *θ *≤ *π *in steps of ∆*θ *= 2π/360.

### Rotating and interpolating the data

After determining the heading direction of the cell data were rotated in order to position the cell parallel to the abscissa and translated such that *C*_90 _was shifted into the origin of the new coordinate system. We denote the axes of the new coordinate system as *x'*-, *y'- *and *z'*- axes and a rotated and translated pixel as , with *j *indicating the number of the pixel in the rotated scan. To determine the lateral extent of the rotated scan we considered the distances of the vertices of the original scan and *y *(*x*, *θ*_h_) or a straight line through *C*_90 _perpendicular to *y *(*x*, *θ*_h_) as illustrated in Figure 4C. Since the approximation of the single line boundaries of the cell soma required lines of data points parallel to the heading direction of the cell, we defined the grid consisting of the projections  of *Q*_*j *_to the *x'*, *y' *plane of the rotated and translated scan such that(4)

Here  is the negative representation of the length  as a coordinate, Δ*x *and Δ*y *denote the step sizes of the original scan in the *x*- and *y*-directions, respectively, and the truncated square brackets represent the *ceil *and the *floor *functions [22,23].

**Figure 5:**
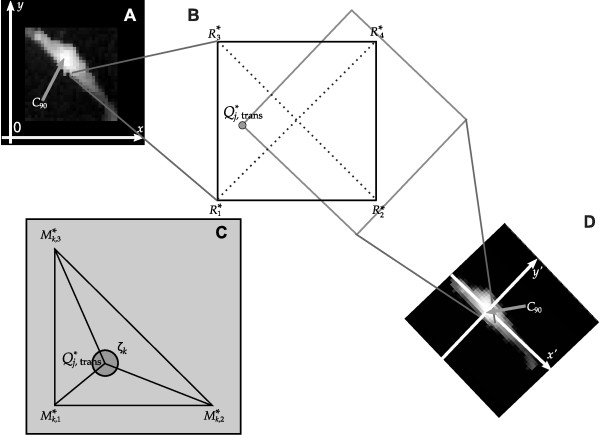
**Interpolation of rotated and translated data**. A: The original data set. B: A magnification of one pixel  of the rotated data and its surrounding four projections of the original data,  to  The triangles **M**_*k *_consisting of three of the projections  are indicated by the dotted lines. D: The rotated data set with *C*_90 _located in the origin. C: The sum ζ_*k *_of the three angles at  to the three points of a triangle is 2*π*.

To obtain the *z'*- coordinate of a pixel *Q*_*j *_we rotated its projection  into the original scan dataset by applying the inverse rotation matrix(5)

and subsequently re-translated it by . We refer to the resulting projection as . If  was located outside the original scan, we defined . Otherwise we considered the four projections  (here  denotes the projection of *P*_*i *_to the *x*-, *y*-plane) that surrounded  as depicted in Figure 5B. The *z*-coordinates of the corresponding pixels were known from the original data. Each set of three out of these four projections defines a triangle as indicated by the dotted lines in Figure 5B. In the following we refer to the four triangles as **M**_*k *_(with *k *= 1, 2, 3, 4) and to the vertices of one triangle as  with *l *= 1, 2, 3. We selected *l *such that the right angle was located at  and furthermore such that  and . An example is shown in Figure 5C. If and only if  was located inside **M**_*k *_the sum ζ_*k *_of the angles at  to the vertices of **M**_*k *_amounted to 2*π *[24].

We next considered the plane defined by the pixels *M*_*k,l *_that corresponded to the projections . The *z*-value *z*_*k*_(*x, y*) of this plane at a position (*x, y*) is given by(6)

We now interpolated  as the average of  if  was located inside **M**_k _:(7)

### Approximation of the contour of a single data row

**Figure 6:**
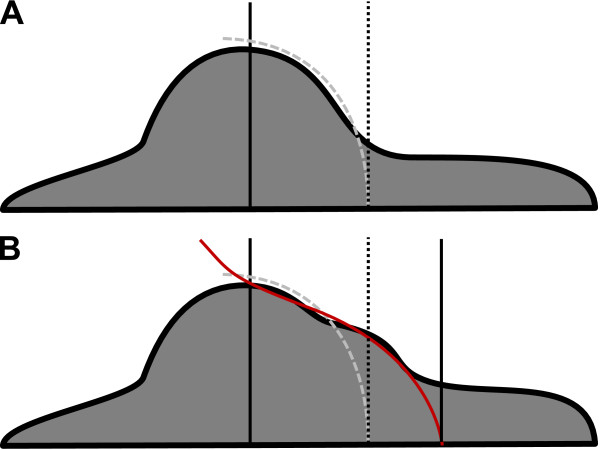
**Characteristic contours of the soma of OPCs**. A: Contour of a cell soma approximating a circular shape. The black line marks the level of *C*_90_. The dashed gray line indicates a parabola fitted to the cell contour that traces the soma but crops the process. B: Contour of a cell soma protruding into the direction of a process. A parabola (gray dashed line) would crop the protrusion of the soma whereas a polynomial of third degree includes the protrusion but still crops the process.

To trace the contour of the cell soma and thus to crop the processes we now considered every data row (all data points with the same *y'*) separately. The corresponding *y'*-values were defined by equation (4). Figure 6 shows sketches of the contours of two characteristic cell shapes; an almost circular cell body that is easy to distinguish from the cell processes (Figure 6A) and a cell soma that protruded into the direction of one of the extensions (Figure 6B). Thus, as indicated in Figure 6B, we assumed that a polynomial of third degree was convenient to approximate the cell soma contour but still suitable to crop the cell process.

To approximate both ends of the cell within a single data row at a fixed *y'*-level we subdivided the data into positive and negative, or frontal and rear, parts with respect to the corresponding *x'*-coordinates. In the following we describe the fitting procedure for the positive part, thus *x' *> 0.  was defined as the projection of *Q*_*j *_to the *x'*, *z'*-plane and furthermore  with *p *= 0, 1, 2,... as the set of projections at a constant *y' *such that for all *p *> 0(8)

Furthermore, we defined  such that . This definition only included pixels with non zero *z'*-coordinates (since the data points were filtered this is equivalent to *z' *> 1 μm, see Methods section). In general *n *+ 2 data points are needed to fit a polynomial of *n*th degree (*n *+ 1 data points define the polynomial). Furthermore, we assumed that the cell body is represented by the data points whose *x'*-coordinates are located close to zero. Thus we additionally tested whether there was no gap within  and it therefore matched the condition(9)

Otherwise, data points with *x'*-coordinates close to zero existed with *z' *= 0. This most likely occured at the borders of the cell soma in ± *y'*-direction and was treated as a special case described later in this section.

**Figure 7:**
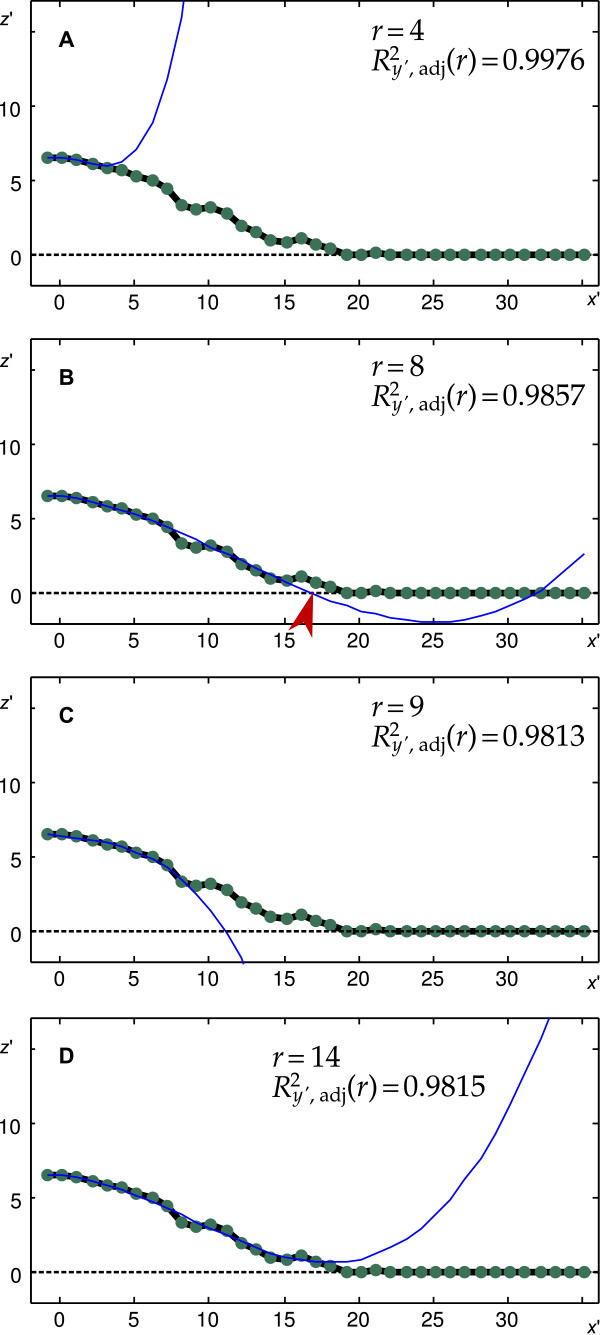
**Example of the fitting procedure**. A-D show the approximated polynomials F _*y' *_(*r*) (blue lines) for *r *= 4; 5; 9 and 14, respectively. Neither *F*_*y'*_(r = 4) nor *F*_*y'*_(*r *= 14) (panels A and D) had a corresponding *X*_*y' *_(*r*) and thus were not taken into consideration. Both *F*_*y' *_(*r *= 8) and *F*_*y'*_(*r *= 9) (panels B and C) had a corresponding *X*_*y' *_(*r*); thus the corresponding  were compared. Since *X*_*y'*_ (*r *= 8) (red arrow-head in B) was selected as the boundary of the cell soma for the investigated *y'*.

To fit a polynomial of *n*th degree to the data we used the function *fit *from Matlab's Curve Fitting Toolbox that implements a least square algorithm [25,26]. It provides, among others, the value  that represents the goodness of the fit considering the number of data points that were approximated by the fit. We investigated the goodness of the fits to an increasing number *r *of data points. We refer to the subset of **S**_*y' *_that contains the first *r *elements as  and we denote the goodness of the fit to **S**_*r,y' *_as  Additionally, we defined *X*_*y'*_(*r*) to be the smallest, positive, non-complex root of the polynomial  that was determined by the function *fit*. We approximated the polynomial boundary of the cell soma for each line segment towards the direction of fitting as the *X*_*y'*_(*r*) that matched the condition(10)

with *r *= *n *+ 1, *n *+ 2,..., *p*_max_. Here *p*_max _denotes the largest index *p *of the projections included in **S**_*y' *_Figure 7 shows examples of the fitting procedure for *r *= 4, 8, 9 and 14, respectively, with *n *= 3, hence fitting polynomials of third degree. For *r *= 4 and *r *= 14 (Figure 7A and 7D) *F*_*y'*_(*r*) had no real root with a corresponding positive *x'*-coordinate, thus these fits were not taken into consideration. Since  (Figure 7B and 7C) *X*_*y'*_(*r *= 8) (indicated by the red arrow-head in Figure 7C) was used to approximate the cell soma boundary at the corresponding *y'*-level. Note that the goodness of the fit to **S**_*8,y' *_was larger than those of all other fits that exhibited *X*_*y'*_(*r *≠ 8) but are not shown in Figure 7 for clarity.

If the procedure failed to determine a cell soma boundary for the investigated set of data points **S**_*y' *_no *r *with a corresponding *X *_*y' *_(*r*) existed. We then defined the boundary to be *X*_*y' *_(*r = n*), if it existed. Note that (*r *= *n*) is not defined [26]. If *X*_*y' *_(*r *= *n*) did also not exist we repeated the procedure with *n *: = *n *- 1 as long as *n *> 1, thus fitting polynomials of a reduced degree. In all cases investigated this procedure led to detection of bordering pixels.

**Figure 8:**
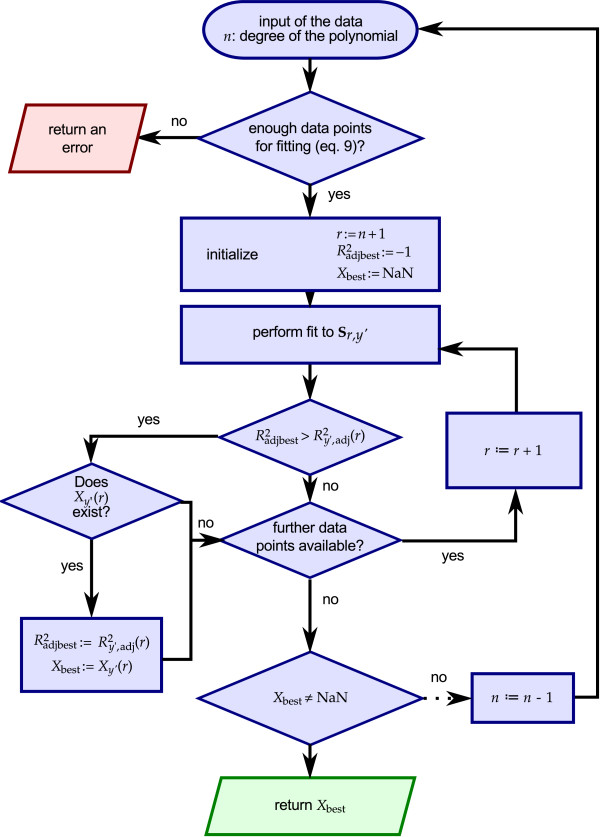
**Flow-chart of the procedure to find the best fit**. The procedure investigates the approximations to an increasing number of data points and selects the one with a positive, non-complex root and the best corresponding  Note that the chart omits some additional tests (see text) to ensure an error free operation as indicated by the dotted arrow in the lower right part. Note that NaN ≡ not a number.

Figure 8 summarizes the fitting procedure as described above in a flow chart. Due to space restrictions the chart omits the test of whether (*r *= *n*) existed as well as the test of whether *n *> 1, indicated by the dotted arrow in the lower right part of the chart. This procedure was named *fitBest*.

#### Special cases of the fitting procedure

As indicated in Figure 8 an error was returned if the investigated set of data points did not match the conditions listed in equation (9). In this case data points with a corresponding  existed within the first *n *+ 1 data points in the fit direction. This most likely occurred at the borders of the cell soma in ± *y'*-direction. This special situation might occur under two conditions. In the first case the cell body approximates to a circular shape causing the boundary perpendicular to the direction of fitting to consist of only a few pixels. Furthermore, the number of pixels available to the fitting procedure as depicted in Figure 7 is decreased by the division of the cell into its frontal and its rear part. Secondly, OPCs in a later stage of development might exhibit small additional extensions that grow perpendicularly to the heading direction.

It was important to consider these cases in order to provide an errorless and thus automatic processing of the data. There are different strategies to determine the boundary of the cell soma at these locations depending either on the chosen degree of the polynomial fitted to **S**_*y' *_as well as whether potential extensions at these sides of the cell soma should be included or excluded from the soma approximation. The most restrictive and simple solution would be to omit and thus to crop these lines.

To obtain a more accurate fit and to include potential cell extensions at these sides we introduced three more functions: *fitOnePoint*, *fitTwoPoints *and *fitThreePoints *that were executed depending on the number of data points with *z' *> 0. We considered the set of pixels  that matched all conditions listed in equation (8) except one: The *z'*-coordinate was not tested, thus **T**_*y' *_might also include projections with *z' *= 0. Let  be the number of projections with a *z'*-coordinate exceeding zero. If *N *= 4 we executed the function *fitBest*. If *N *= 1, *N *= 2 or *N *= 3 we executed the functions *fitOnePoint*, *fitTwoPoints *or *fitThreePoints*, respectively. Note that these functions might result in more than one boundary for the particular *y' *level, thus the resulting approximated cell soma might appear jagged.

The simplest case is *N *= 1 and the corresponding function *fitOnePoint*. We refer to the non-zero data point as  and used the roots of a parabola through  as the boundary if *u *< 4, otherwise the line was cropped.

Let the two non-zero projections be  and  with *u *<*v *in the case of *N *= 2 (function *fitTwoPoints*). We first considered the case *v - u *= 1, hence, the two points were neighbors. We fitted a polynomial of third degree to  and  and used its roots as the boundary in this case except if *v *= 4 and . In the latter case we cropped the structure assuming that it did not belong to the cell soma.

If *v *- *u *> 1 we only assigned  to the cell soma and approximated the contour of the cell soma by the roots of the parabola through  as in the function *fitOnePoint*.

The most complicated case was *N *= 3. We refer to the single projection with zero *z'*-coordinate as  In this case the approximation was performed differently for varying values of *u*. If *u *= 1 we considered the *z'*-coordinate of the projection . If  we assumed that the cell soma exhibited an asymmetric shape and applied the function *fitBest*. Otherwise, if  we approximated the cell soma boundary for the particular *y' *by the roots of a polynomial of third degree fitted to .

If *u *∈ {2, 3} we applied *fitOnePoint *to the single, non-zero projection and *fitTwoPoints *to the two neighboring non-zero projections, respectively. If *u *= 4 we considered the *z'*-coordinate of the first point opposite to the direction of fitting, . If  we applied *fitBest*, otherwise we approximated the cell soma boundary at the current *y'*-level by the roots of a polynomial of third degree fitted to .

### Approximation of the volume of the cell soma

To approximate the cell soma volume we summed the *z*-coordinates of every pixel located within the approximated boundaries of the cell soma. This required that the height of every pixel located within the approximated cell soma boundary was known. Hence, if a single delimitation of the cell soma was located outside the original scan we were not able to approximate the cell soma volume and the recording was discarded. This happened if the cell body was in part located outside of the SICM image or very close to its borders.

### Evaluation of the procedure

To evaluate the BDA we simulated objects of known volume and applied the morphometric fitting procedure to investigate any potential effect of geometry on the volume determinations. We have previously determined the restrictions of scan size and resolution for the successful investigation of migrating OPCs [27]. In brief, to image migrating OPCs with a suitable frame rate using our present SICM the dimensions of the recordings had to be restricted to 30 μm squares with a lateral step size of 1 μm, limiting the SICM images to 900 pixels.

**Figure 9:**
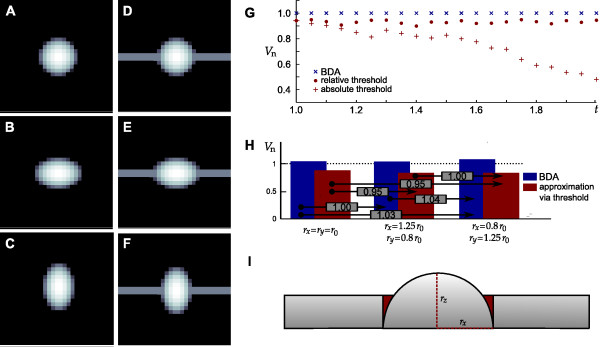
**Application of boundary delimitation algorithm to simulated objects**. A-C: Half-ellipsoids with the corresponding radii *r*_*x *_= *r*_y _= *r*_0 _(A), *r*_*x *_= 1.25 *r*_0_; *r*_y _= 0.8 *r*_0 _(B) and *r*_*x *_= 0.8 *r*_0_; *r*_*y *_= 1.25 *r*_0 _(C). The radius in *z*-direction is *r*_*z *_= *r*_0_. D-F: Hemisphere/half-ellipsoids from A-C with additional extensions. G: Normalized volume (*V*_n_) computed by the boundary delimitation algorithm (BDA) as well as by thresholding simulating a half-ellipsoid with the radii *r*_*x *_= *t r*_0 _and *r*_*z *_= 1/*t r*_0 _for 1 ≤ *t *≤ 2 and Δ*t *= 0.05. Corresponding thresholds were 0.4 *r*_*z *_and 0.4 *r*_0_, respectively. H: Volumes of the objects from D-F computed by the BDA (blue) and by thresholding (red) using a threshold of 0.4 *r*_z_. Gray boxes indicate erroneously determined changes in volume when the shape of the object changes as indicated by the respective arrows. I: The addition of the extensions changes the volume of the simulated cell soma with respect to the mere half ellipsoid as indicated by the red area.

We first applied the BDA to a hemisphere with a radius of *r*_0 _= 5 pixels (since the length of the cell body of an OPC is approximately 10 μm) in a data set consisting of 900 pixels as depicted in Figure 9A. The volume *V*_comp _computed by the BDA (omitting the determination of a heading direction as well as rotation and translation) was the same as the volume *V*_sum _calculated by summing the volume of the columns above each pixel.

We next compared the determination of the volume of an half-ellipsoid with the two methods. A possible effect of the direction of fitting was tested by applying the BDA to an ellipsoid defined by the three radii *r*_*x*_, *r*_*y *_and *r*_*z *_with *r*_*x *_> r_y _and vice versa, as depicted in Figure 9B and 9C (the corresponding radii are *r*_*x *_= 0.8*r*_0_, *r*_*y *_= 1.25*r*_0_, *r*_*z *_= *r*_0 _and *r*_*x *_= 1.25*r*_0_, *r*_*y *_= 0.8*r*_0_, *r*_*z *_= *r*_0_). Again, no difference was found between *V*_comp _and *V*_sum_.

To investigate whether the BDA in principle allows one to determine the volume of an object that flattens but maintains its volume by a compensatory widening we computed the volumes of an ellipsoid defined by the radii *r*_*y *_= *r*_0_, *r*_*x *_= *t r*_0 _and *r*_*z *_= *r*_0_/*t *with 1 ≤ *t *≤ 2 in step sizes of Δ*t *= 0.05. Figure 9G (blue crosses) shows the computed volume normalized to *V*_sum _for every investigated value of *t*. There is no difference between *V*_comp _and *V*_sum_, thus *V*_*n *_= 1. In contrast, the computed volume did not match *V*_sum _when it was determined by using the method that every pixel exceeding a predefined threshold was assigned to the cell soma [16,19]. The normalized volumes are displayed in Figure 9G (red dots and cross-hairs) for an absolute and a relative threshold. In the following we only consider the determination using a relative threshold since it is clearly visible that the use of an absolute threshold leads to increasing differences in the determination of the soma with increasing elongation of the ellipsoid. Additionally, we observed no difference in the volume determined by the BDA and *V*_sum _when varying *r*_*y *_instead of *r*_*x *_or when varying both lateral radii by defining *r*_*x *_= *r*_*y *_= *t*^*1/2 *^*r*_0_.

To simulate a bipolar cell we added extensions in ± *x'*-direction to a hemisphere of radius *r*_0 _as well as to the ellipsoids. Images of the resulting objects are depicted in Figure 9D-F. The height of the extension was chosen as *r*_0_/2 and its width as 2 *r*_0_/5. Every *z*-value at the corresponding positions was adjusted to *r*_0_/2 if the *z*-value calculated by equation (11) (see Methods section) was below *r*_0_/2. This avoids a gap between the half-ellipsoid and the extension but also increases the *z*-value of some pixels of the half-ellipsoid such that the volume differs from the volume *V*_sum _computed by summing the *z*-values of the mere half-ellipsoid as depicted in Figure 9I. To our knowledge no exact definition exists describing where the cell soma ends and the cell process starts. At positions where the soma merges into the neurite a gradual decline of the soma and a corresponding increase of soma volume most likely occurs (Figure 9I).

Here we chose to use the calculated volume of the half-ellipsoid without extension as reference. Since we calculated the soma volume by summing all *z*-values corresponding to pixels within the approximated soma boundary an overestimation of the soma volume at positions merging into the neurites (Figure 9I) could be induced by the BDA.

The approximated volume, normalized to *V*_sum _of the corresponding hemisphere or ellipsoid without extension, is shown in Figure 9H. As expected, the BDA (blue bars in Figure 9H) overestimates the volume with respect to *V*_sum_. In contrast, the approximation via the threshold method [16,19], in this case applied using a threshold of *r*_0_/2, underestimates the volume with respect to *V*_sum _since it omits all sections of the ellipsoid with a height below the selected threshold. Putative cell shape changes as depicted in Figure 9E-G would result in detections of relative soma volumes as indicated in the gray boxes in Figure 9H. Erroneous changes due to different shapes are indicated by the arrows. Both methods lead to almost similar errors (about 5%) in the determination of soma volume changes.

**Figure 10:**
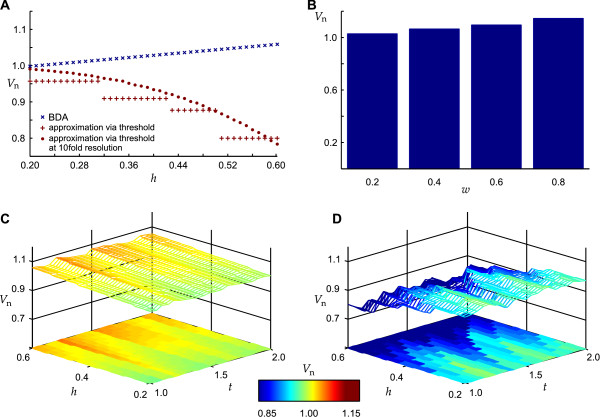
**Simulating objects with varying geometrical parameters**. A shows the volume normalized to the corresponding *V*_sum _determined by the boundary delimitation algorithm (BDA, blue crosses) and via thresholding (red dots) when simulating the height of the processes as *h r*_*z *_with Δ*h *= 0.01. The stepwise decrease vanishes when the resolution increases (red cross-hairs). B shows the impact of the width of the processes by simulating their widths as *w *2 *r*_*y *_. Note that only the results for the BDA are shown. C and D show the volume normalized to the corresponding *V*_sum _when simulating objects with changing the processes' height (as in A) and the radii of the half-ellipsoid as *r*_*x *_= *r*_*y *_= *t*^1/2 ^*r*_0 _and *r*_*z *_= 1/*t r*_0_. The color coded area in the bottom indicates *V*_n _corresponding to the color bar plotted between C and D. C shows the results for the BDA, D for thresholding. The corresponding steps of the parameters were Δ*h *= 0.01 and Δ*t *= 0.05.

Since it is unlikely that the shape of the soma changes while the extensions maintain their shape we next investigated the impact of changes in the shape of the extensions. Figure 10A shows the volume determined by the BDA when applied to a hemisphere with adjacent extensions (as depicted in Figure 9E) of varying relative height *h r*_0_, normalized to *V*_sum _of the hemisphere without extensions. As expected from the result shown in Figure 9H, our method overestimates the volume with increasing height. An increase in the height of the extension from *h *= 0.2 to *h *= 0.6 results in an erroneous detection of a soma volume increase of about 6%. Although the relation seems to be linear in the depicted range, it is more complex: A threefold increase in the height of the extension from *h *= 0.3 to *h *= 0.9 leads to an erroneous detection of a volume increase of about 9% whereas a threefold increase from *h *= 0.1 to *h *= 0.3 leads to an erroneous detection of a volume increase of about 3%. In contrast, the thresholding method (red cross-hairs) shows an underestimation of the soma volume that increases stepwise but maintains a constant volume over a range of heights. However, the stepwise decrease of the calculated volume and thus the determination of a constant volume over a certain range of heights results from the imprecision that occurs due to the rasterization of the sphere as shown by the investigation of a simulated scan with a tenfold resolution (red dots in Figure 10A). An increase in the height of the extension from *h *= 0.2 to *h *= 0.6 results in an erroneous detection of a volume decrease of about 16% for the low resolution simulation and of 32% in the high resolution simulation. We observed similar results when performing the same investigation on the objects depicted in Figure 9F and 9G with only slight differences in the amount of errors determined by the two methods.

We used *h r*_0 _as threshold in these investigations. Note that the height of the processes of a live cell is much more difficult to determine due to the more complex and irregular shape and thus adds additional uncertainties to the determination of the soma volume.

Figure 10B shows the impact of various widths of the extension on the soma volume determination. As expected, the overestimation of the soma volume increases with increasing extension width *w *2 *r*_0_. A fourfold widening of the extension leads to an erroneous determination of a soma volume increase of 11%. Since the height of the extension defines the threshold for the thresholding method the increasing width of the extension is not detected by this method. Thus it computes a constant volume under these conditions.

Figure 10C and 10D show the impact of a combined variation of the radii and the height of the extension. We investigated the radii *r*_*x*_(*t*) = *r*_*y*_(*t*) = *t*^1/2 ^*r*_0 _and *r*_*z*_(*t*) = *r*_0_/*t *for 1 ≤ *t *≤ 2 and the fraction *h *of the height *h r*_z _(*t*) of the extension for 0.2 ≤ *h *≤ 0.6. Particularly when minor changes in shape were simulated, the BDA (Figure 10C) detects smaller erroneous volume changes compared with the thresholding method (Figure 10).

### Application to live cells

**Figure 11:**
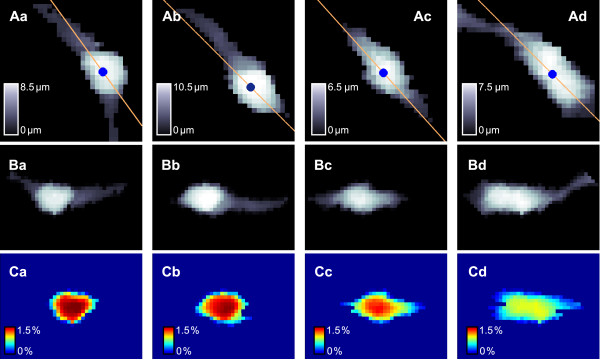
**Application of the boundary delimitation algorithm to live cells**. A: Top views of the original data of four different OPCs obtained by pulse-mode SICM in floating backstep configuration. Orange lines indicate *y *(*x*, *θ*_h_), blue dots indicate *C*_90_. B: Top views of the rotated, translated and interpolated data from the corresponding scans from A. The height of each scan and its corresponding rotated data is indicated by the color bars in A. C: Approximated soma area of the corresponding scans. The contribution of each pixel to the entire soma volume is indicated by the color bars at the bottom of each panel.

We next applied the BDA to determine soma volumes in SICM recordings of live cells that exhibited both a much more irregular shape than the simulated objects as well as extensions that might be more difficult to distinguish from the cell soma. The corresponding data is available as Additional File 2. Figure 11 shows the results of the BDA applied to four different OPCs from rat brain. Note that the cells were positioned along the diagonal of the scan field in order to include as many details of the cell ramifications as possible. Whereas the cell somata depicted in Figure 11 Aa and Ba approximate a circular shape the OPCs shown in Figure 11 Ca and Da exhibited a more elongated cell soma that merged into one of the processes. The determination of the heading direction of the OPC shown in Figure 11 Aa selected the direction of the major process. Note that this might not be true when the fraction of the minor process that is located within the scan area notably exceeds the fraction of the major process. This might not impair the determination of the cell soma from a single scan but might have an impact when investigating the soma volume of a cell that migrates along the major process.

**Figure 12:**
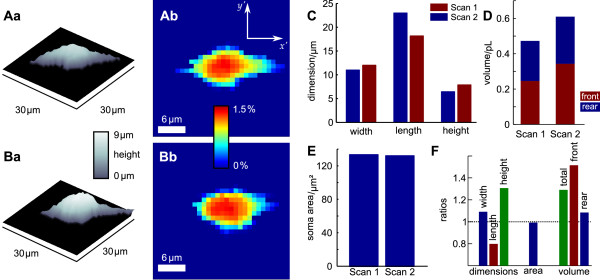
**Shape and volume dynamics of the soma of a migrating cell**. A and B show three dimensional representations of successive SICM recordings at a time interval of 11 min and the corresponding boundary delimitation approximations of the cell soma. Color bars indicate height and the contribution to the entire soma volume per pixel, respectively. C shows the corresponding widths, lengths and heights of the cell soma. Whereas the width remained constant, the length decreased and the height increased. D shows the corresponding soma volumes, subdivided into frontal and rear part with respect to the direction of migration at the level of *C*_90_. Note the swelling of about 140 pL. E shows the size of the area covered by the cell soma. Note that in contrast to the cell soma volume, the area covered by the soma slightly decreased. F shows the ratios of the determined geometrical parameters illustrating their changes between the two scans.

Figure 11B shows color coded representations of the rotated, translated and interpolated data sets. It is clearly visible that the transformed data faithfully represent the original data. Figure 11C shows the approximated basal area of the cell soma and the relative contribution of each pixel to the entire cell soma. A considerable difference becomes visible between the circular and the elongated cell somata. In the latter case the major part of the expansion into the process is assigned to the cell soma. The reason for this becomes more apparent in the three dimensional representation of an OPC exhibiting this type of soma shape as depicted in Figure 12A. At the left side of the SICM image the soma merges smoothly into the process. Hence, it is a comprehensible interpretation to assign this part of the cell to the cell soma.

In the second recording the cell changed its shape to be more circular and thus the determined basal area of the cell soma only shows a slight expansion as clearly visible by the comparison of Figure 12Ab and 12Bb. Note that it is known that migrating OPCs show an average velocity of 10 μm/h [28] and that migrating OPCs show notable changes in shape [27]. The detailed analysis of the parameters of the cell shape and soma shows that the cell swelled by approximately 29% and that this swelling was dominated by an increase in cell height whereas the length of the soma decreased. Most notably, this challenges the method to approximate the volume of a cell from light microscopic images by interpolation via the width and the length of its soma. This approximation, in contrast, would detect a slight cell shrinkage since the basal area covered by the cell soma was reduced as depicted in Figure 12E. The separate consideration of the frontal and rear soma volume by dividing the cell soma at the level of *C*_90 _perpendicular to *y *(*x*, *θ*_h_) yields that the volume increase is dominated by an increase in the frontal volume (Figure 12E). Figure 12F summarizes the changes in the lateral dimensions as well as the changes of area and volume between both scans.

## Conclusions

We provide a new algorithm to approximate the basal area of the cell soma for volume determinations of bipolar cells undergoing temporal changes in shape. Simulations show that this method provides smaller errors in the detection of soma volume changes in moving cells than the method to separate soma areas from neurites via height thresholds [16,19]. We show that the algorithm can be applied successfully to detect soma volume changes of living oligodendrocyte precursor cells. Additionally we demonstrate that the approximation of the volume using two-dimensional data such as cell body length and width may lead to erroneous results.

## Methods

### Scanning ion conductance microscopy

Cells were obtained as described in [27] and images were acquired using a pulse mode SICM operating in floating backstep mode as detailed in [15,29]. Scans were performed in Leibovitz-15 medium using scanning probes with an access resistance of about 4 MΩ. Scanning probes were filled with extracellular saline containing (in mM): NaCl 110, KCl 5.4, CaCl_2 _1.8, MgCl_2 _0.8, HEPES 10, glucose 10. Step sizes were 1 μm in lateral and 100 nm in vertical direction, frame acquisition time was about 10 minutes.

### Determination of the position of the nucleus

Staining of the nucleus was performed by applying Hoechst 33342 dye (20 μg/mL, in Leibovitz-15 medium) for 5 minutes before the SICM measurement was started. Fluorescence images were obtained with a Zeiss Axiovert microscope at an epifluorescence wavelength of 365 nm. The boundaries of the nucleus were obtained by applying the Sobel algorithm and thresholding. Centroids were calculated in the same manner as described above (equation (1)) but using a color value as threshold. The position of the scanning area within the light microscopic image was determined as described previously [19].

### Data processing

SICM data was processed using Matlab (R2008a) including the Curve Fitting Toolbox [25]. Data shown were interpolated by cubic splines unless otherwise noted. All *z*-data were plane corrected if necessary and filtered with a threshold filter setting each *z*-value less than 1 μm to 0 μm before the BDA was applied.

### Creation of half-ellipsoids

The *z*-coordinates of the half-ellipsoids were computed as(11)

Here the operator ℜ was defined to return the real part of a complex number *a *+ *b*i, hence *a *= ℜ(*a *+ *b*i) [22] and *r*_*x*_, *r*_*y *_and *r*_*z *_denote the radii of the ellipsoid in the corresponding directions.

### Availability and requirements

**Project name: **BDA - A boundary delimitation algorithm

**Project homepage: **https://sourceforge.net/projects/behindlight/files/BDA/

**Operating system(s): **Platform independent

**Programming language: **Matlab

**Other requirements: **Matlab R2008a with Curve Fitting Toolbox

**License: **GNU-LGPL

**Restrictions for commercial usage: **Notify the corresponding author of this report and include a citation to this report in the accompanying documentation.

## Authors' contributions

PH and IDD designed the research. PH, RK and IDD wrote the manuscript. PH developed and coded the approximation method, KM performed the SICM measurements and the light microscopic determinations of the nucleus, prepared the cell culture and participated in the preparation of the manuscript. All authors read and approved the final manuscript.

## Supplementary Material

Additional file 1 **This file is a zip archive including the Matlab code of the algorithm described above**. To access the BDA extract (e.g. by using the unzip tool) the archive into a directory included in Matlab's path or, preferably, adjust Matlab's path. The archive consists of the following files (a detailed description is included in the respective file): **cellDirection.m **Determines the heading direction of a cell. See eqs. (2) and (3). **CellFit.m **Class performing the fitting procedure. See eqs. (8), (9) and (10), figs. 7 and 8 and section *Approximation of the contour of a single data line*. **CellSomaVolumeCalculation.m **Main class performing the BDA. It calls the respective procedures and class constructors and provides access to the data as described below. It calculates the dimensions of the rotated scan, see fig. 4 and eq. (4). **centroid2 D.m **Calculates the centroid of a given area, see eq. (1). **getAreaAtHeight.m **Reduces the *z*-coordinates of a set of pixels to boolean values indicating whether the *z*-coordinate exceeds a threshold. See section *Approximation of the position of the nucleus*. **getInterpolZ.m **Calculates the interpolated *z'*-coordinate, see eqs. (6) and (7). **getLineFromCentroid.m **Calculates the *x'*-, *y'*-, - and *z'*-coordinates of a line parallel to the heading direction of the cell at a selectable distance from the *C*_90_, therefore it performs the rotation and translation of the data. See eq. (5). **SICMScan.m **Class file for reading SICM data. To apply the BDA to scanning probe microscopical data stored in a text file consisting of three columns representing the *x*-, *y*- and *z*-coordinates use cell = CellSomaVolumeCalculation('*</path/to/data>*') Here, *</path/to/data>*has to be substituted with the path and filename of the data to be investigated. After the BDA has finished the methods cell.plot, cell.plotRotated and cell.plotVolume are available to plot the raw data, the rotated and interpolated data and the contribution of each pixel to the cell soma volume, respectively (see fig. 11). The properties cell.Vol, cell.frontVol and cell.rearVol contain the total, front and rear volume, respectively (note that front and rear in this case represent +*x *and -*x*-direction), cell.centroid and cell.angle contain the coordinates of *C_*_*T*_ (as described above, we used *T *= 0.9; the threshold can be varied in the properties section of CellSomaVolumeCalculation.m) and the heading direction of the cell, respectively. Click here for file

Additional file 2 **This file is a zip archive containing the data of the cells depicted in figs. **11**and **12. Note that these data were processed as described in the Methods section. Files within the archive are named according to the figure label. Since the data shown in Figure 11Ac and Figure 12A originate from the same recording we included a soft link instead of a copy of the data. Click here for file
